# Essentials of Homœopathic Therapeutics

**Published:** 1895-12

**Authors:** 


Essentials of Homœopathic Therapeutics, being a Quiz Compend upon the application of homœopathic remedies to diseased states. A companion to the *Essentials of Homœopathic Materia Medica.* Arranged and compiled especially for the use of students of medicine by W. A. Dewey, M. D. Philadelphia: Bœricke & Tafel, 1011 Arch Street, 1895. Price, cloth, $1.50; by mail, $1.58; half morocco, $1.75; by mail, $1.83.

As stated above, Dr. Dewey has compiled a previous handy volume entitled *Essentials of Homœopathic Materia Medica,* which was reviewed in this journal for April, 1894, at page 125, to which the reader is referred. The volume now under notice is a companion to it, and contains the names of the diseases, arranged in alphabetical order, with questions upon the most suitable remedies for these disorders and the indications electing them. Of course, every question is followed by an answer, and the student who will resolutely memorize the contents of this book will find himself possessed of a very fair knowledge of the universal application of drugs to diseases with which to begin practice. Upon this foundation he can then begin to rear an elaborate superstructure of materia medica, which, sooner or later, will bring him to the front as a first-class physician.

It cannot be too strongly impressed upon the student that, as is so forcibly said in the preface, "one of the grand cardinal features of Homœopathy, and one little understood by the allopathic school, is the fact that any drug in the entire homœopathic materia medica may be a remedy in any diseased state." This feature is, we think, but indifferently understood by the majority of our own school, and it cannot too often be dwelt upon by all who assume to teach the principles of Hahnemann.

